# The Brain Matures with Stronger Functional Connectivity and Decreased Randomness of Its Network

**DOI:** 10.1371/journal.pone.0036896

**Published:** 2012-05-15

**Authors:** Dirk J. A. Smit, Maria Boersma, Hugo G. Schnack, Sifis Micheloyannis, Dorret I. Boomsma, Hilleke E. Hulshoff Pol, Cornelis J. Stam, Eco J. C. de Geus

**Affiliations:** 1 Biological Psychology, VU University, Amsterdam, The Netherlands; 2 Clinical Neurophysiology, VU Medical Centre, Amsterdam, The Netherlands; 3 Neuroscience Campus Amsterdam, VU University, Amsterdam, The Netherlands; 4 Department of Psychiatry, University Medical Center Utrecht, Utrecht, The Netherlands; 5 Clinical Neurophysiology Laboratory L Widén, University of Crete, Iraklion, Greece; 6 EMGO+ Institute, VU Medical Centre, Amsterdam, The Netherlands; Cuban Neuroscience Center, Cuba

## Abstract

We investigated the development of the brain's functional connectivity throughout the life span (ages 5 through 71 years) by measuring EEG activity in a large population-based sample. Connectivity was established with Synchronization Likelihood. Relative randomness of the connectivity patterns was established with Watts and Strogatz' (1998) graph parameters C (local clustering) and L (global path length) for alpha (∼10 Hz), beta (∼20 Hz), and theta (∼4 Hz) oscillation networks. From childhood to adolescence large increases in connectivity in alpha, theta and beta frequency bands were found that continued at a slower pace into adulthood (peaking at ∼50 yrs). Connectivity changes were accompanied by increases in L and C reflecting decreases in network randomness or increased order (peak levels reached at ∼18 yrs). Older age (55+) was associated with weakened connectivity. Semi-automatically segmented T1 weighted MRI images of 104 young adults revealed that connectivity was significantly correlated to cerebral white matter volume (alpha oscillations: r = 33, p<01; theta: r = 22, p<05), while path length was related to both white matter (alpha: max. r = 38, p<001) and gray matter (alpha: max. r = 36, p<001; theta: max. r = 36, p<001) volumes. In conclusion, EEG connectivity and graph theoretical network analysis may be used to trace structural and functional development of the brain.

## Introduction

The brain is a complex network of highly connected brain areas that exchange information via long-range axonal projections. Several methods are available to investigate connectivity. Anatomical methods include Diffusion Tensor Imaging tract tracin [1], [2] and connectivity derived from cortical gray matter thicknes [3], [4]. Functional methods use direct (EEG, MEG) or indirect (fMRI BOLD) measures of correlated neuronal activity to derive networks of functionally coupled brain areas. fMRI is a measure with a high spatial resolution which has been found to consistently extract subnetworks in the brain that show activity modulation on a time-scale of tens of seconds (Resting state network [5], [6]). MEG/EEG, on the other hand, may be used to estimate short duration networks that arise and disappear on a scale of seconds, although it has been shown that both types of resting state networks share a common groun [7], [8].

In addition to establishing overall brain connectivity, graph theoretical analysis allows the evaluation of whole-brain efficienc [9] and the determination of network topologies, such as small-world network [10]–[12]. Networks can be classified as ordered, small world, or random by the use of two graph parameters, clustering coefficient C and path length [13], [14]. Watts and Strogat [12] showed that highly ordered networks (high C) with only a few random links could achieve optimal connectivity (short L) close to the random state. These small-world networks have favorable properties with efficient information transfer and resilience to (simulated) attack [10], [15]–[17]. Confirming this evolutionary advantage, human brains were shown to have this small-world topolog [15], [18], which indeed favors cognitive performanc [19], [20]. Interestingly, people differ systematically in the graph parameters and these differences are geneti [21], [22]. In addition, deviant graph parameters have been found in disease states compared to control [23]–[25] which might therefore act as markers for developmental psychopathology or neurodegeneration.

**Figure 1:**
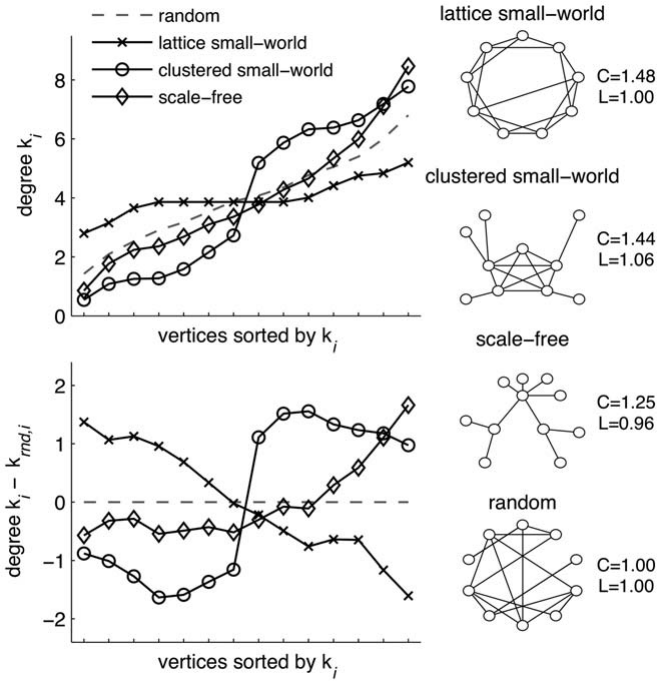
Degree distributions of prototypical networks. Right column shows illustrations of prototypical networks: the (ring) lattice small-world, the clustered small-world, and the scale-free network. Note that all ordered networks have small world properties as C>1.0 and L∼1.0, albeit to a different degree. Random networks serve as baseline for all comparisons (C = 1.0 and L = 1.0). From the three types of ordered networks, graphs (number of vertices: N = 14, average degree  =  average number of connections per vertex: K = 4.0) were simulated with three random reconnections to ensure small-world properties. The resulting degree distribution holds the number of connections sorted low to high (Top left plot) with the dashed line representing the average of 1000 randomized graphs. The three types of ordered graphs show highly distinctive degree distributions when plotted relative to the random graph (bottom left plot). Lattice small-world networks have relative flat degree distribution (around 4) resulting in a negative slope when compared to random networks. Clustered small-world networks have one set of vertices with low degree, another with high degree resulting in a rotated-S-shaped curve. Scale-free networks have a exponentially increasing curve in their degree distribution.

Small-world networks can be topologically subdivided in different subtypes. Figure 1 shows three prototypical networks that all show path lengths close to random networks, while showing high levels of clustering compared to random networks–the hallmark of small-world network [11], [12]. Watts and Strogat [12] based their initial analysis on the first subtype, the one-dimensional lattice small-world graph, which has a flat degree distribution with most vertices having the same number of connections. The second subtype, here termed clustered small-world graphs, show a highly uneven degree distribution with a collection of highly (inter)connected hubs. The brain may well have such a neocortical cluster of hubs in adulthoo [1], [15], but it is unclear whether this cluster is present from childhood on or develops with age. Finally, scale-free graphs show also show an uneven degree distribution, but with exponentially increasing number of connections. This graph subtype shows the highest level of skew in the degree distribution, and may be less optimal evolutionary because of the vulnerability to targeted attack [14].

Although the graph parameters C and L make statements about network quality along the dimension of random – small world – ordered, they may not reveal these qualitative topological differences and their development over time [26]: All of the above networks, although in different degrees, show small-world properties of a relatively high C and short L. This topological network quality may be revealed by inspecting the number of connection each vertex (node) has: the *degree distribution* K  =  {k_1_, k_2_, …, k_N_} where k_i_ holds the number of connections for each vertex i = <1, N> sorted from low to high. Inspection of the degree distribution of networks in different age groups can reveal these qualitative changes–such as the development of cortical hubs–thus providing insight into the underlying growth rules underlying the neural network developmen [27].

In this study, we used EEG in relative short (12 s) periods to establish synchronization between EEG signals from relatively distant brain areas, thus reflecting long-range connectivity. We did this in a sample spanning a large age range (5 to 71) so that we could chart the development of functional connectivity and degree of order assessed from graph parameters across the life span. As a second aim we tested whether the brain network changes its topological quality over time, in addition to its degree of order. Brain maturation involves marked changes in anatomical structure, including an initial increase into childhood and subsequent continuous decrease in gray matter volume as well as density, the protracted increase of white matter volume, and decline of fractional anisotrop [28]–[33], as a result of synaptic density changes (pruning), myelination, and axonal diameter change [34]. As a final aim we investigated whether brain anatomy was correlated with the observed differences in functional connectivity–and the graph parameters derived from these networks–by correlating these with cerebral white matter volume (WMV) and gray matter volume (GMV) established from MRI scans available in a young adult subset of the subjects. Although restricted to one age group, a correlation between functional connectivity, network randomness, and underlying anatomical variables may prove helpful in understanding how the observed large changes in brain anatomy–including both young developmen [30], [34], [35] and agin [33], [36], [37]– shapes brain activity and, ultimately, brain function.

## Methods

### Subjects and procedure

Data were collected as part of an ongoing study into the genetics of brain development and cognition. A total number of 1675 individuals (twins and additional siblings) accepted an invitation for extensive EEG measurement. For the present analyses, EEG data recorded during 3–4 minutes of eyes-closed rest were available from six measurement waves with ages centered around 5, 7, 16, 18, 25, and 50 years. Part of these consisted of longitudinal measurements at two ages (5–7 and 16–18 years). In addition, some of the subjects aged 16–18 years were invited back for measurements at age 25. In total, this study incorporated 2540 EEG recordings. After data cleaning, 2137 datasets were available. The structure of the final subject set after data cleaning used in the present study was 331, 368, 418, 380, 350, and 290 for the six measurement waves, which included 294 longitudinal observations between 5 and 7, 374 between 16 and 18, 96 between 18 and 25, of which 95 with measurements at three waves 16, 18, and 25.

Ethical permission was obtained via the "subcommissie voor de ethiek van het mensgebonden onderzoek" of the Academisch Ziekenhuis VU (currently named METc of the VUmc). All subjects (and parents/guardians for subjects under 18) were informed about the nature of the research. All subjects or parents/guardians were invited by letter to participate, and agreement to participate was obtained in writing. All subjects were treated in accordance with the Declaration of Helsinki.

### EEG acquisition

The childhood and adolescent EEG were recorded with tin electrodes in an ElectroCap connected to a Nihon Koden PV-441A polygraph with time constant 5 s (corresponding to a 0.03 Hz high-pass filter) and lowpass of 35 Hz, digitized at 250 Hz using an in-house built 12-bit A/D converter board and stored for offline analysis. Leads were Fp1, Fp2, F7, F3, F4, F8, C3, C4, T5, P3, P4, T6, O1, O2, and bipolar horizontal and vertical EOG derivations. Electrode impedances were kept below 5 kΩ. Following the recommendation by Pivik et al.[38], tin earlobe electrodes (A1, A2) were fed to separate high-impedance amplifiers, after which the electrically linked output signals served as reference to the EEG signals. Sine waves of 100 μV were used for calibration of the amplification/AD conversion before measurement of each subject.

Young adult and middle-aged EEG was recorded with Ag/AgCl electrodes mounted in an ElectroCap and registered using an AD amplifier developed by Twente Medical Systems (TMS; Enschede, The Netherlands) for 657 subjects and NeuroScan SynAmps 5083 amplifier for 103 subjects. Standard 10–20 positions were F7, F3, F1, Fz, F2, F4, F8, T7, C3, Cz, C4, T8, P7, P3, Pz, P4, P8, O1 and O2. For subjects measured with NeuroScan Fp1, Fp2, and Oz were also recorded. The vertical electro-oculogram (EOG) was recorded bipolarly between two Ag/AgCl electrodes, affixed one cm below the right eye and one cm above the eyebrow of the right eye. The horizontal EOG was recorded bipolarly between two Ag/AgCl electrodes affixed one cm left from the left eye and one cm right from the right eye. An Ag/AgCl electrode placed on the forehead was used as a ground electrode. Impedances of all EEG electrodes were kept below 3 k**Ω**, and impedances of the EOG electrodes were kept below 10 kΩ. The EEG was amplified, digitized at 250 Hz and stored for offline processing.

### EEG preprocessing

We selected 14 EEG signals (Fp1, Fp2, F7, F3, F4, F8, C3, C4, T5, P3, P4, T6, O1, O2 and both EOG channels) for further analysis. For subjects without Fp1 and Fp2 recordings, these were substituted with their closest match F1 and F2. The reason for this lead replacement was that a reduced (12 lead) graph yielded graph parameters much closer to random values, with a reduced power to detect age differences. This substitution was tested by comparing 46 unrelated individuals who had both F1, F2 and Fp1, Fp2 sets available. The correlations *r*(C_F1F2_, C_Fp1Fp2_), *r*(L_F1F2_, L_Fp1Fp2_), and *r*(SL_F1F2_, SL_Fp1Fp2_) were very high (.93>r>96 for alpha oscillations, .75 <r<.97 for beta oscillations). Even though correlations were high, the C, L, and SL scores showed a small but systematic bias (<.043 for C, <054 for L, and <15 for SL). This bias was removed in all subsequent scoring.

All signals were broadband filtered from 1 to 37 Hz with a zero-phase FIR filter with 6dB roll-off. Next, we visually inspected the traces and removed bad signals. Note that for the network analysis a full set of EEG signals was required and therefore any rejected EEG channel resulted in the loss of that subject. Next, we used the extended ICA decomposition implemented in EEGLA [39] to remove artifacts, including eye movements, and blink [40]. After exclusion of components reflecting artifacts, the EEG signals were filtered into the alpha (6.0 to 13.0 Hz) and beta (15.0 to 25.0 Hz) frequency bands. The peak alpha frequency developed from 8.1 Hz at age 5 to 9.9 Hz at age 18, after which a slow decline to 9.4 Hz was observed at around 50 years. The lower edge of the alpha filter was set such that alpha oscillation of all subjects was included from ∼2.0 Hz below the lowest peak frequency to ∼3.0 Hz above the highest peak frequency. EEG power in the defined theta, alpha, and beta frequency bands was determined using Welch' method on 50% overlapping stretches of 4096 samples.

### Connectivity

EEG signals are thought to reflect the neural activity of the brain tissue that results from synchronous dendritic input across a large cortical area. Connectivity was calculated using synchronization likelihood (SL) following Stam and van Dij [41]. SL is based on generalized synchronization between coupled systems removes the overestimation shown by coherence in filtered signals, and detects linear as well as non-linear connectivity. In short, if a signal s1 is in a certain state (to be defined below) at time i we may find a recurrence of that state at another time point j. Next, we look if the second signal s2 is in the same state at time points i and j, recording a hit if so. SL is defined as the proportion of hits (in s2) to the total number of recurrences (in s1) and is thus a number between 0 and 1. Note that SL is found even when signals s1 and s2 are in different states, as long as s1 and s2 are self-similar at i and j.

More formally, the instantaneous state of an EEG signal was represented by m-dimensional state vectors **X**_i_  =  {x_i_, x_i_ + 1l, x_i_ + 2l, …, x_i_+(m−1)l } where l is the lag and m the embedding dimension. The elements of **X**_i_ are m samples taken from the signal spaced l apart. The vector is taken to represent the state of the system at time i. Within the same signal recurrences are sought at times j that reflect a similar state: A threshold distance ε is chosen such that a fixed proportion (p_ref_  = 0.02) of comparisons are close enough to be considered in a similar state. Next, the same comparison is made for a different system Y at the same time points i and j and with the same value for p_ref_. Now the synchronization likelihood S_i_ between X and Y at time i is defined as follows:
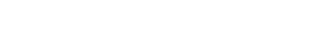
where **θ** is the Heaviside step function returning 0 for all values <0 and 1 for values > = 0. Time point j is chosen with a minimum lag from i and depends on the lower value of the filter, so as to avoid autocorrelation effects. N represents the number of recurrences of the state **X_i_** within X. Overall SL between X and Y is the average over all possible i. The settings for SL calculation were those recommended by Montez et al.[42]. Note that these settings are not critical in the calculation of S [21].

### Graph analysis

Graphs were created by thresholding the SL matrices such that the total number of (bidirectional) connections in the graph was 32 resulting in an average number of connections per vertex (node) of K = 4.0. Graph parameters clustering coefficient C and path length L were calculated following Watts and Strogat [12]. In short, C is calculated for each vertex as the proportion of neighboring vertices that are interconnected between them. That is, if vertex v1 is connected with v2 and v3, this constitutes a closed triangle, and an open triangle if they are not. C is then n_closed_/(n_closed_+n_open_). Overall graph C is the mean across all C_i_, i = <1, N>. Path length L is also calculated for each vertex, and reflects the average minimum number of steps required go from the current vertex to all other vertices, passing only along existing edges. Note that the averaging procedure for L is the harmonic mean L = 1/sum(1/Li) with unconnected nodes assigned the value of +∞. This reduces the influence of unconnected nodes while retaining the full network siz [11]. All C and L scores were normalized to reflect deviation from randomness by dividing each score with the average of C and L from 1000 Erdös-Rényi random graphs with the same number of vertices and average degree as the empirical graphs. A value of 1.0 therefore reflects a value as in the random case.

Note that for subjects with replaced leads (F1, F2 in stead of Fp1, Fp2) we removed the systematic bias as explained above.

### Structural MRI Assessment

From 104 subjects (62 male; average age 27.4 years) magnetic resonance imaging (MRI) scans were aquired at a Philips 1.5 T Intera scanner (Philips, Best, The Netherlands) at the University Medical Center Utrecht. For a detailed description of the aquistion and processing of the scans of this sample, see Baaré et al. [43]. In short, the T1-weighted images (voxelsize 1×1×1.2 mm^3^) were transformed into Talairach orientation (no scaling) [44] and corrected for magnetic field inhomogeneitie [45]. Segments of gray and white matter of the cerebrum were obtained by an automated method validated earlie [46], from which tissue volumes were calculated.

We calculated partial correlations (accounting for sex differences) between MRI volumes and the EEG parameters. As SL and L were highly skewed we applied a log transformation and a −1/x transformation respectively (note that the negation keeps the direction of the correlation intact) to effectively normalize the distributions of these variables. C was approximately normally distributed. Bootstrap resampling was used to calculate confidence intervals for the partial correlations (see statistics).

### Statistics

Observations were split into nine age groups with age boundaries in years: 4.9 – 6.0, 6.0 – 7.4, 15.0 – 17.0, 17.0 – 20.0, 20.0 – 25.0, 25.0 – 35.0, 35.0 – 45.0, 45.0 – 55.0, and 55+. These groups were labeled ∼5, ∼7, ∼16, ∼18, ∼22, ∼30, ∼40, ∼50, and 55+. Final group sizes and average ages are shown in Table 1.

**Table 1:** Age group definition and size.

Age group (yrs)	N	Mean (SD)	Range
∼5 (below 6.0)	331	5.3 (0.19)	(4.93–5.86)
∼7 (6.0–7.5)	368	6.8 (0.19)	(6.45–7.46)
∼16 (15.2–17.0)	447	16.1 (0.47)	(15.23–16.99)
∼18 (17.0–20.0)	345	17.6 (0.39)	(17.00–18.92)
∼22 (20.0–25.0)	148	23.4 (0.90)	(20.21–24.95)
∼30 (25.0–35.0)	176	28.6 (2.52)	(25.04–34.55)
∼40 (35.0–45.0)	96	41.6 (2.33)	(35.40–44.99)
∼50 (45.0–55.0)	149	48.9 (2.44)	(45.32–54.26)
55+(55.0 and older)	55	60.8 (4.14)	(55.35–71.03)

Because the complex structure of the data including repeated measures and family dependencies, which even extended across the different age groups (siblings of twins might fall into a different age category than the proband twins), we established significance via bootstrapping. The bootstrap consisted of randomly selecting (with replacement) from the pool of families, retrieving the data for all family members, and calculating the statistic (i.e., the difference in group means) and estimating its confidence interval. Sampling on the family level rather than individual level keeps–on average–the complex covariance structure between the family members and repeated measures intact. Confidence intervals were adjusted using the bias correction and accelerated metho [47], and a conservative alpha level of .01 was used. Group differences tested were limited to adjacent age groups and groups two steps apart.

## Results

### Protracted development of connectivity

**Figure 2:**
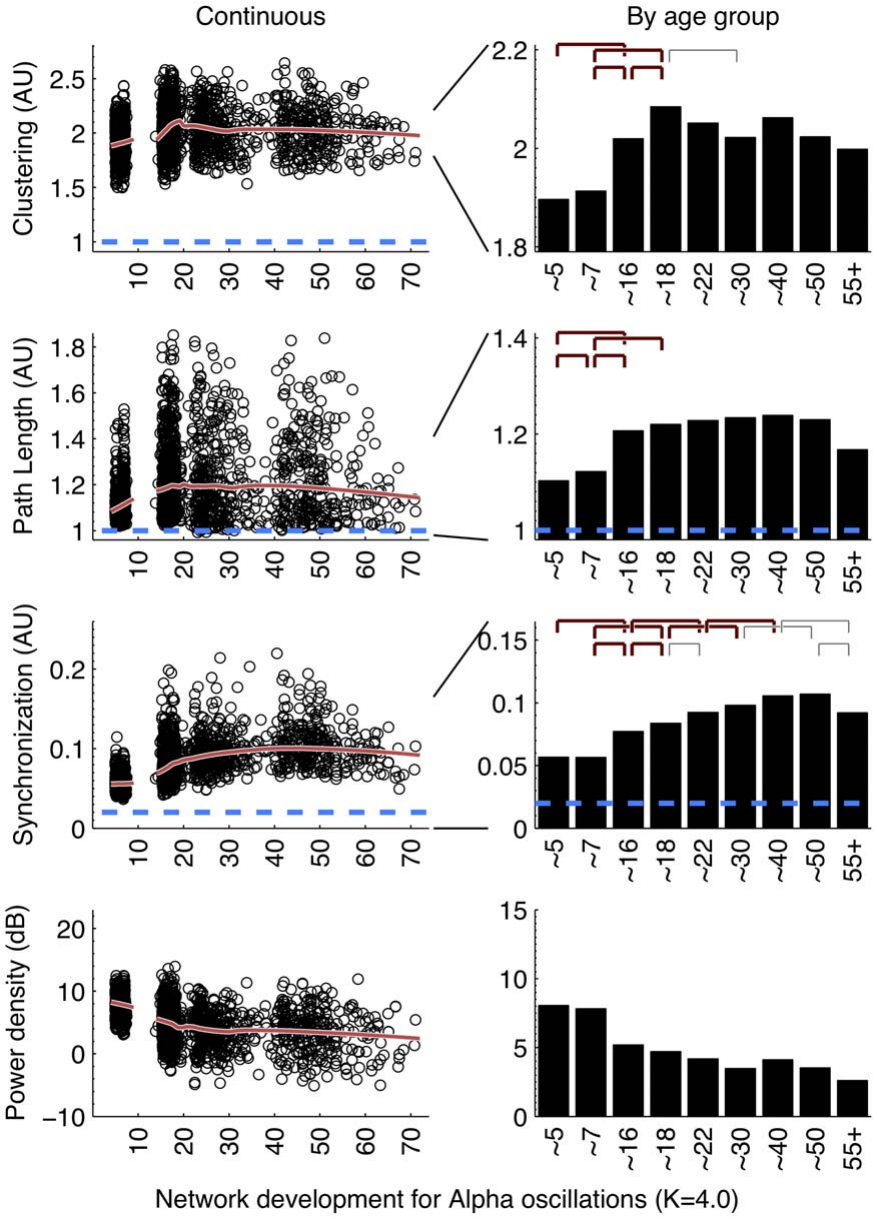
Alpha band (6 –**13 Hz) development of Clustering, Path Length, average connectivity, and average power.** SL is the average connectivity of synchronization likelihood across all possible pairs of signals. Clustering C and Path Length L were obtained as indicated in the text. Dashed lines are values obtained for randomized networks (C and L) or connectivity between two white noise signals (SL). (Left column:) Each variable is plotted with a continuous predictor and a quadratic loess smooth 40% of the data. Results show a small-world organization throughout life as L∼1.0 while C>>1.0. Large changes were observed for L, overall connectivity, and less so for C. The opposite development of the power of alpha oscillations suggests that increased order is not a spurious effect of increased signal-to-noise ratios. (Right column:) Means by age group. Hooks indicate bootstrap determined significant difference (gray: p<.01, thin black p<001, thick black p<0001) between adjacent and next-adjacent groups. Significant increases between childhood and adolescence and even within adolescence (for C) indicate a decrease of brain network randomness with age. A stable period for C after ∼18 and for L after ∼16 yrs was observed. Overall connectivity significantly increased up to ∼40 but peaked at ∼50, and showed a significant decrease into older age (55+).

**Figure 3 pone-0036896-g003:**
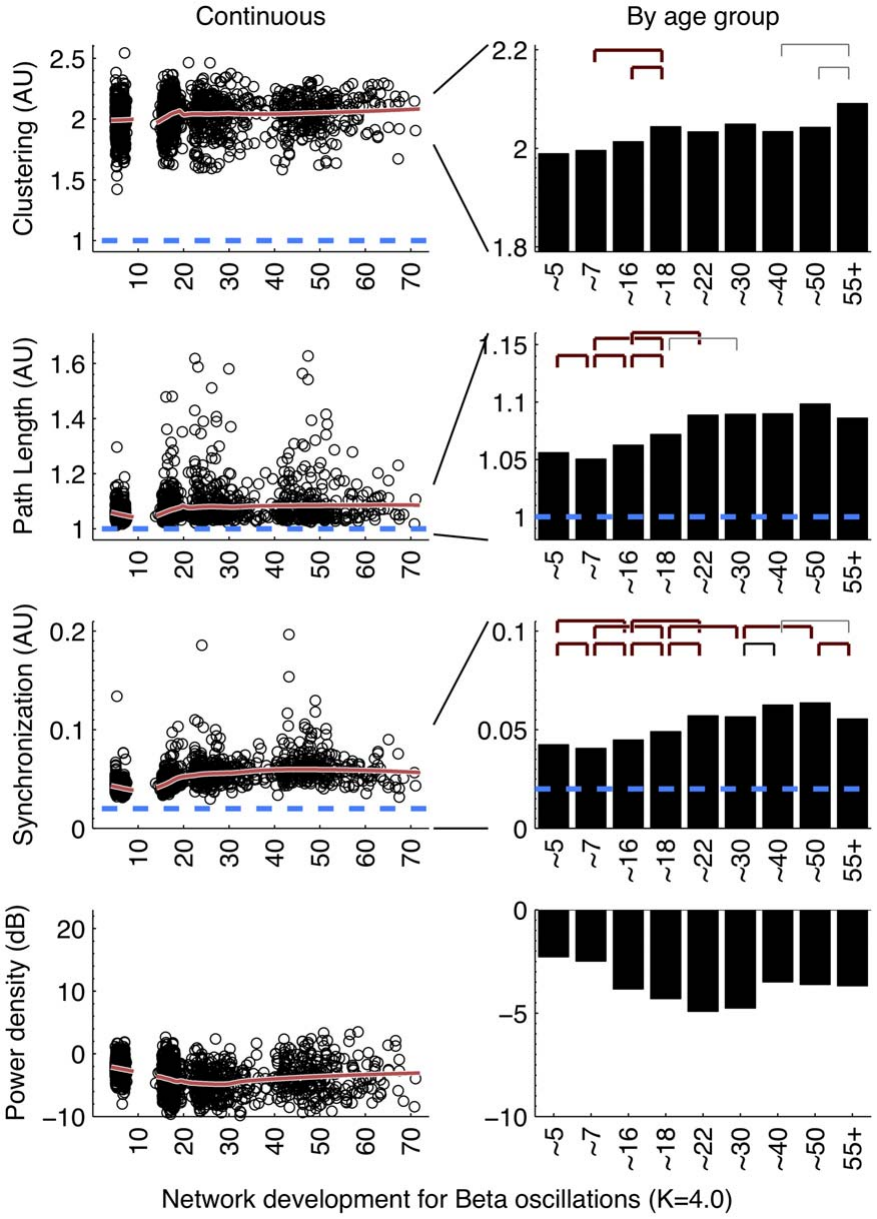
Beta band (15 –**25 Hz) development of parameters C and L, average connectivity, and average power.** See figure 2 for additional legend. (Left column:) Results are highly similar to alpha oscillatory networks, including a small-world organization throughout life and an opposite development of the power suggesting that increased order is not a spurious effect of increased signal-to-noise ratios. (Right column:) Bootstrap showed significant increases between childhood and adolescence and even within adolescence (for both C and L) indicate increased brain network order. A stable period for C and L after ∼18 yrs was observed. Overall connectivity significantly increased up to ∼40 but peaked at ∼50, and showed significant decrease into older age (55+).

**Figure 4:**
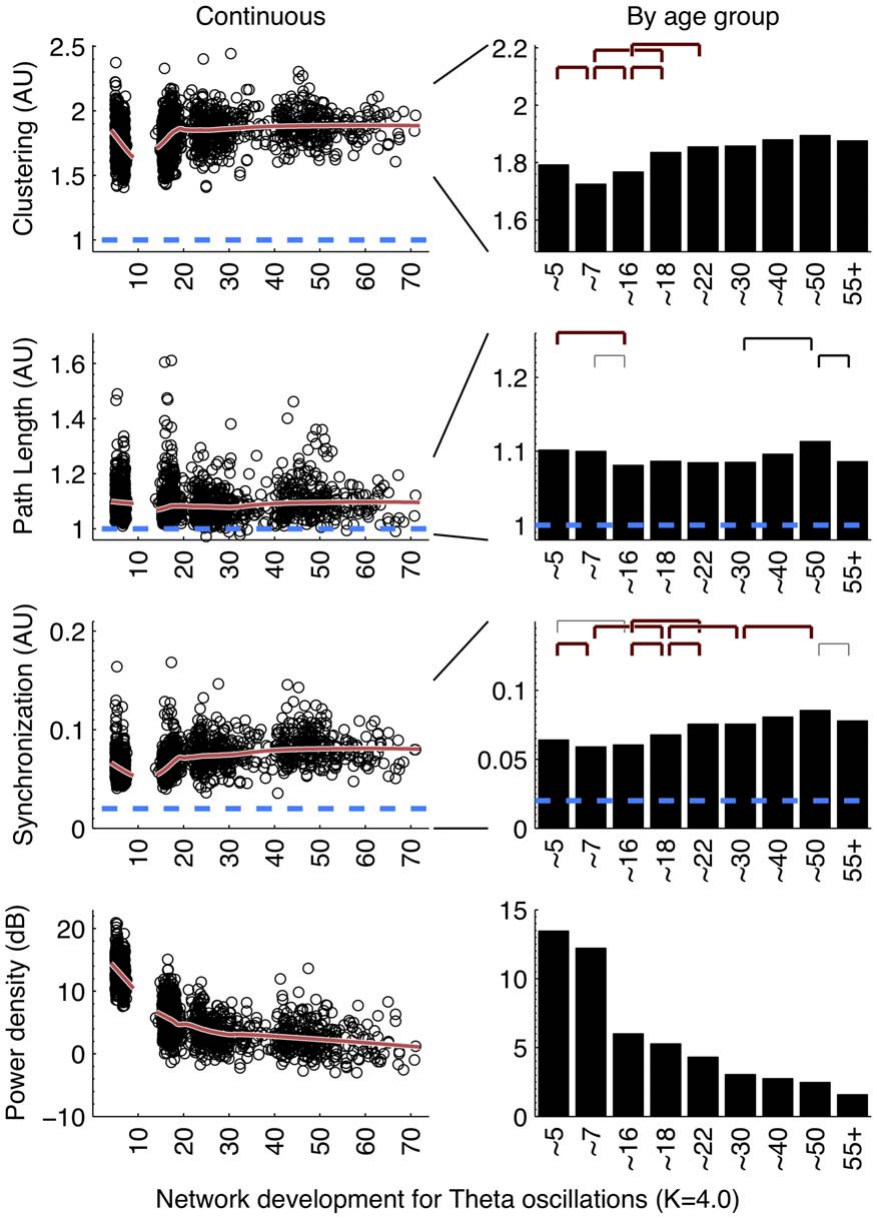
Theta oscillations (3.0–5.6 Hz) development of parameters C and L, average connectivity, and average power. See figure 2 for additional legend. (Left column:) Childhood ages showed elevated levels of all parameters including theta power. (Right column:) Bootstrapping showed significant increases between childhood and adolescence for C and SL. L and SL additionally showed an increase between ∼30 and ∼50 yrs, and a subsequent decrease to 55+.

All three frequency bands (Figures 2, 3, and 4) showed similar development of synchronization likelihood (SL) which significantly increased with age reflecting the prolonged maturation of brain connectivity. The connectivity enhancement extended well into adulthood and even into middle-aged adulthood, after which a plateau developed. All three figures show that peak connectivity was found at age ∼50. A significant decline in connectivity was only first observed in the 55+ age group for all frequency oscillations.

### Graph parameters revealed large increases in network order between childhood and adolescence and decreases in late adulthood

Overall, the development in C and L shows a change in the quality of the brain network from a relative random to a more ordered organization, from childhood to adolescence. Alpha and beta frequency bands showed evidence for increases in both C and L between childhood and adolescence (Figures 2, 3, left columns). Significance of these changes is indicated in the right columns. C additionally shows a clear increase within adolescence. In adolescence, a plateau is reached, which is by-and-large maintained throughout adult life. Only beta band C shows a significant increase in the oldest age group. Development of theta networks in early childhood deviated from this pattern, as there was an initial decrease in connectivity and graph parameter C from age 5 to 7. This is most likely related to the very large decrease in theta power during the first decade of life. Significant increases within adulthood years were observed for L (∼30 to ∼50), reflecting a relative stability between adolescence and adulthood for this measure.

Network development in the first two decades of life reach maximal levels at the age of ∼18 for C and L. Older age was generally associated with decreases in both C and L but these effects were only significant for L in the theta band. A significant increase in C was found for networks from beta oscillations.

**Figure 5:**
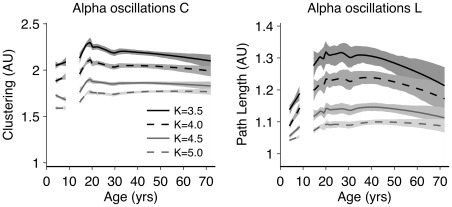
Average degree K marginally influences the developmental profiles of graph parameters C and L. C and L development from connectivity matrices of alpha (6–13 Hz) oscillations are shown. A loess smooth is surrounded by 95% confidence intervals. Marked differences can be seen in the developmental plots, most prominent is the steady decrease in both C and L (relative to the random case) with increased K with a relatively intact curvature; this shape–not the absolute level–reveals the development and comparison between age groups. Some of these age effects were consistent across levels of K: 1) An increase during adolescence in both C (about 16 to 19 yrs) and L (about 16 to 20 yrs)–indicating that brain development is not finished until young adulthood and provides evidence for in increasingly ordered brain networks; 2) Childhood levels of C and L are lower than adolescent and young adult levels. Developmental profiles also differed across levels of K: Adult ages showed a decline in C (for K<4.5) and a strong decline in L (for K<5.0), indicating increased random network organization.

Figure 5 shows the development of the graph parameters C and L (alpha oscillations only) as a function of average degree K chosen in the thresholding procedure. Many age effects were independent of the choice of K. This indicates a certain generality of the effects and an independence of the arbitrary choice of K. Childhood is marked by highly random network connectivity patterns, that slowly increased to more ordered networks. For all levels of K, the changes observed between age groups 5 and 7 were minimal compared to the increase in order from childhood to adolescence. Adolescence also showed increase in order of the brain network, but the maximal values for C are reached somewhat earlier than for L. Finally, older age is characterized by a reversal towards increased randomness.

### Connectivity and network change are unlikely to be caused by spurious power effects

It could be argued that a lack of connectivity between brain areas–and a resulting random network configuration–could be the result not of the absence of connectivity per se, but the absence of oscillations from which they are derived. Since EEG power density reflects the amount and strength of the oscillations, the spurious effect would predict a positive relation between EEG power and connectivity (or the graph parameters derived from it). Figures 2 to [Fig pone-0036896-g003] 4 (bottom rows) show that EEG power density of both alpha and beta oscillations follow a very different developmental trajectory than the connectivity parameters. In general, they are in the opposite direction of the spurious effect (i.e., a decrease in power is associated with an increase in C and L, i.e. a depart from randomness), giving no support to this alternative explanation. One notable exception is the strong decrease in theta power associated with concurrent decreases in C and L. Therefore, concurrent changes in theta power may have caused the deviant patterns for theta connectivity in the childhood age groups.

### Topological network quality is stable for alpha, theta oscillations but changes for beta oscillations

**Figure 6:**
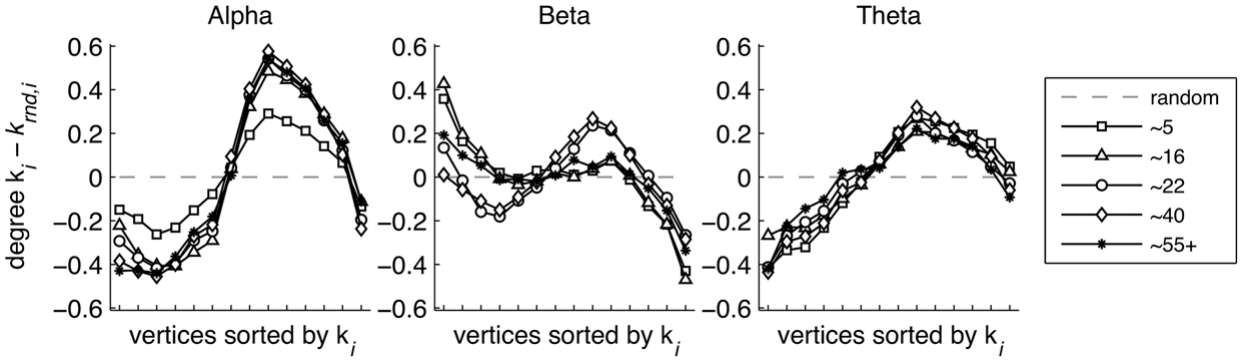
Stability and change in network quality. Sorted degree distributions relative to random graphs for 5 different age groups can be matched to prototypical networks described in figure 1. Alpha oscillation networks (left plot) consistently showed a clustered small-world organization. Childhood ages showed a degree distribution closer to random, but the clustering is clearly visible. Increased age created increased deviation from random but did not qualitatively change the network. Beta networks (right plot) showed a degree distribution close to random for children and adolescents, but since the clustering coefficient was much larger than 1.0 (figure 3), networks in these groups are perhaps best described as a mix between lattice and clustered small-world network. The brain progressed into a clustered small-world network in adulthood (∼22 to ∼50 years) but increased tilt again for the eldest groups (55+) indicating a more lattice small-world network. Theta oscillation networks also showed evidence for a clustered small-world network with a set of nodes with high degree, and a set of nodes with low degree. This pattern was observed throughout life.

Topological network quality was next assessed by inspecting the degree distribution K_i_ of the brain connectivity graphs, sorted low to high and averaged over all epochs/subjects. These were plotted for age groups ∼5, ∼16, ∼22, ∼40, and 55+ years (Figure 6), and can be visually compared with the degree distributions obtained for the three prototypical ordered graphs of Figure 1. For graphs from alpha oscillations, it was clear that the networks represented a clustered small-world network throughout life, as evidenced by the S-shaped degree distribution. Interestingly, the S-shape did not qualitatively change, but increased in amplitude, suggesting that the connectivity within the cluster becomes more pronounced as age progresses and reaching a plateau already at age ∼16. Therefore, the degree distribution of alpha oscillation networks shows no evidence for qualitative topological change from ∼5 to ∼55 years of age.

A similar clustered topology was obtained for graphs from theta oscillations. Although the nodes with lowest degree were somewhat lower than expected, the sorted degree distributions consistently showed high degree for a subset of nodes and low degree for the remainder, reflecting the core property of a clustered network. The topology was quite stable across age groups.

For beta band oscillations, networks represented a flatter degree distribution than a clustered small-world network with even a slightly downward slope–an indication of a lattice small-world network. Childhood, adolescent, and the oldest age groups showed the flattest degree distribution–an indication of increased randomness. This provides some evidence that the beta oscillatory network is in an immature state before adulthood with an underdeveloped network cluster. This cluster only reaches full interconnectivity between the hubs in the cluster at ∼22 years of age. Older age is associated with increased randomness and deterioration of the cluster.

### SL and L correlate with white and gray matter brain volume in young adult subjects

In 104 young adults, the relationship was tested between EEG-based connectivity and graph parameters on the one hand and white and gray matter volumes assessed by anatomical scans using MRI on the other. Table 2 shows the resulting partial correlations (accounting for sex) both for the full sample and after removing highly influential subjects: two with abs(z-score)>4.0, and one male subject with small brain volume and unusually large effect on the confidence intervals. Significant correlations between SL and WMV were observed (theta: r = 22, p<01; alpha: r = 33, p<01). No significant effects between SL and GMV. Correlations between brain volumes and graph parameter L showed moderate effect sizes. L was correlated with both WMV (theta: max r = 36, p<001; alpha: max r = 38, p<001) and GMV (alpha: max r = 36, p<001), fairly independent of choice of threshold K (although networks with K = 3.5 were too sparse to reliably detect effects). C did not significantly correlate with the brain volume parameters, albeit only just in some cases (alpha: max r = 22, p<05).

**Table 2:** Partial correlations between cerebral volumes and functional measures (connectivity SL, graph parameters C and L) corrected for sex.

				C				L			
MRI volume	EEG frequency	SL	*K* =	3.5	4.0	4.5	5.0	3.5	4.0	4.5	5.0
*All subjects*											
GMV	Theta	0.02		0.04	0.09	0.07	0.02	0.06	0.12	0.16	0.01
	Alpha	0.18		0.11	0.15	0.13	0.08	0.26+	0.33*	0.32*	0.29*
	Beta	0.04		0.04	0.00	0.06	0.05	0.04	0.04	0.03	0.00
WMV	Theta	0.21*		−0.01	0.05	0.10	0.03	0.17	0.28*	0.34**	0.24**
	Alpha	0.27+		−0.08	0.00	0.14	0.22+	0.21	0.26+	0.26+	0.28*
	Beta	0.16		0.10	0.10	0.09	0.13	0.05	0.07	0.06	0.09
*After removing extreme values*											
GMV	Theta	0.02		0.05	0.10	0.08	0.02	0.07	0.13	0.16	0.02
	Alpha	0.23+		0.11	0.15	0.14	0.09	0.26+	0.34*	0.36**	0.32*
	Beta	0.05		0.04	0.01	0.07	0.06	0.03	−0.06	−0.02	0.03
WMV	Theta	0.22*		0.00	0.06	0.12	0.03	0.19	0.29*	0.36**	0.25**
	Alpha	0.33*		−0.08	0.02	0.16	0.24+	0.25+	0.34*	0.37**	0.38**
	Beta	0.18		0.11	0.12	0.12	0.15	0.08	0.10	0.07	0.09

Note. GMV = gray matter volume, WMV = white matter volume, K = threshold on connectivity matrix such that average degree K is reached, SL  =  average synchronization likelihood, L = Path Length, C = Clustering Coefficient. SL and L were log and −1/x transformed to normalize the distributions. Significance was determined via bootstrap resampling of families and confidence intervals estimated with the bias-corrected and accelerated method. Lower table: Two subjects with >4.0 SD deviation on SL, L, or C, and one male subject with very small intracranial volume were removed. **p<.001, *p<.01, +p<.05 (trend).

## Discussion

The main aim was to investigate the development form childhood to adulthood of the strength and patterning in long-range connectivity. For this, we estimated connectivity based on synchronization likelihood (SL) between EEG signals from distant electrodes for a large sample aged 5 to 71 years. Average SL showed large increases from childhood to adolescence. Previous reports suggested that EEG connectivity reflects (maturational processes of) white matter tract properties. For example, interhemispheric EEG connectivity (coherence) has been related to DTI diffusivity in localized bundle [48], and to T2 relaxation times in both white and grey matter which may be related to neuronal membrane lesion in head injur [49] but may also reflect maturatio [50]. The view that functional connectivity measured with SL reveals properties of the underlying white matter is supported on several grounds.

First, there is a highly suggestive correspondence between the protracted development of SL and the development of WMV as reported in the extant literature. Peak levels of SL were found at age ∼50 for theta, alpha, and beta oscillations, while a significant decrease in connectivity was found only in later life (55+). These results are highly consistent with reported peak ages for WMV development in large sample studies (peak at ∼44 yrs for frontal lobe WM [37], ∼48 yrs for temporal lob [37]; 50.1 for whole brai [33]; ∼38 for whole brai [31]; ∼43 for whole brai [32]; ∼38 in female WM [51]), although some reports could not establish significant (nonlinear) trends in WMV development (in male [51]) or reported regional specificity in the peak age [37], [52]. In addition, WMV increases reflect ongoing myelination, which shows a similarly protracted development (50–59 yr [53]).

Second, in a modestly sized subsample of young adults we observed positive correlations between SL and MRI-derived WMV for the oscillations across the three frequency bands (3.0–25.0 Hz), which reached significance for the slower oscillation networks (3.0–13.0 Hz). Although this result is limited to a single age group of about 27 years, it suggests that SL may index differences in adult WMV, the underlying developmental processes of myelination leading up to adult WMV, and its functional determinants or effect [54]. Given the importance of oscillations in large-scale networks for cognitive processin [55], it can be hypothesized that functional connectivity mediates this link between WMV and cognition by increasing communication and coordination between distant brain areas. In addition, our results support the notion of EEG connectivity as a biomarker for developmental psychopathology. For example, autism has been related to both increased white matter and long range EEG connectivit [54], whereas callosal white matter loss has been related to decreased cross-hemispheric connectivity in Alzheimers' diseas [56].

Besides average connectivity strength, we investigated the pattern of connections using a graph theoretical approach. To this end, we applied Watts and Strogatz' [12] approach to estimate C and L to the thresholded connectivity matrices. Contrary to previous findings on the graph theoretical analysis of functional connectivity networks (fMR [57], EE [58]), we found strong increases in both C and L from childhood to adolescence as well as within adolescence. This was found for all oscillations (3.0–25.0 Hz). Concurrent increases in C and L are an indication of decreased network randomness and increased orde [59,60]. Importantly, the developmental trend in the network parameters, e.g. L in the alpha band, leveled off at a much earlier age than SL, suggesting that it provides complementary information to SL on the development of the brain network. The pattern of developmental changes in network order was fairly independent of the choice of threshold degree K. Across K levels, maximal values of C and L were reached earlier (∼18 yrs) than for SL. Although the basic networks seem to be in place from a very early age (Figure 6)[57], [61]–[63], the increased order in the brain network suggests that they nonetheless differ in randomness, and therefore in essential computational capacities between childhood and adolescenc [64]. This is consistent with the idea that maturation reflects ongoing functional segregation of consistent networks that are decreasingly diffus [63] and therefore less random.

A striking finding was that–in the theta band–the significant decreases in connectivity in older age (55+) were accompanied by decreases in L, suggesting that brain tissue atrophy results in changes in the brain network. Decreases in L are hypothesized to reflect efficiency in information transfe [10], [15]. The neural loss observed in later lif [65], [66] may well be causative of this element of reduced order in the brain network, and could perhaps be seen as a non-clinical variant of the disrupted networks found in Alzheimers' Diseas [17], [67].

Graph parameter L was positively correlated to WMV in an adult sample. In addition, developmental profiles of both L and WMV (as reported in the literature) show marked increases from childhood to young adulthood. As with SL, these results suggest that L is predictive of white matter development. This was expected, since we have previously shown that L and SL are correlated measure [68]. However, it was somewhat unexpected that L (for alpha oscillation networks) positively correlated to GMV. Thus, L carries additional information about functional brain connectivity that is not covered by average connectivity strength per se, but is reflected in the efficiency of the network organization. The continuous decline of GMV in adolescence and adult lif [30], [37] is thought to reflect the degree of synaptic pruning and connective trimmin [28], [61], [69]. The observed correlation suggests that these pruning processes decrease network efficiency, possibly by strengthening connectivity within subnetwork [63]. Note that it remains unclear how the positive correlation within the young adult age group relates to the observed developmental paths, so that firm conclusions cannot be drawn. Even so, the data clearly suggest that L may be used to chart normal gray matter development and psychopathology that is associated with abnormal gray matter development, such as schizophrenia. Biomarkers of schizophrenia include changes in the prefrontal cortex caused by reduced neuropil (assumed to reflect loss of connectivity), reduced spine densities, and smaller dendritic arbor [70], [71], thus resulting in GMV abnormalities. Indeed, graph theoretical analysis of EEG and fMRI resting state activity has shown deviant networks in this diseas [71], [72].

The current results revealed increased connectivity for alpha and beta band oscillations, but make no distinction between long and short range connectivity. Short range connectivity requires much denser electrode placement, which is likely to result in spurious connectivity from volume conduction effect [73]. Recent observations have suggested that the dichomotmy in projection length is essential, and yields opposite results: decreased short range connectivity concurs with increased long-range connectivity with age. In an fMRI study it was shown that local activity in cognitive control networks becomes less diffuse with age, which is accompanied by increased long distance functional connectivit [74]. Similar findings of changes in (long-distance) connectivity have been reporte [56], [61], [75]. The present results extend these findings in showing that from childhood to adulthood brain networks move from random to ordered. Since network parameters are relevant predictors of cognitive performanc [19], [20], and are disrupted in psychopathology [58], we can hypothesize that the increased order is essential to the large developmental changes in human cognitive performance during the same period. This may be addressed in future investigations.

Topographical network quality–assessed by inspecting the sorted degree distribution of the graphs and comparing these to three prototypical network types (Lattice small-world, Clustered small-world, and Scale-free)–showed evidence of clustered small-world networks for many age groups and oscillation frequencies. A clustered small-world network shows a group (or more than one group) of hubs, consistent with a modular organization observed in resting state fMR [76]. Similarly, evidence for clusters of hubs has been presented in networks in both adults and infant [77]. The current results showed that this network quality is relatively stable over a wide age range (for alpha and theta oscillation networks), but shows some development for networks derived from beta frequency oscillations. The shift in tilt in the sorted degree distribution for age groups ∼5, ∼16 and 55+ indicates that younger age have an immature cluster of hubs that only becomes fully operational at the age of ∼22 years, and deterioration of that cluster in older age.

In conclusion, we have shown that brain maturation across the lifespan may be tracked using inexpensive EEG recordings. The brain showed protracted increases in connectivity consistent with white matter developmental curves, and changed from a relatively random to a more ordered configuration. The EEG-derived individual differences in connectivity and efficiency of the brain's connectivity network reflected actual anatomical differences as assessed by MRI. Since the network parameters used here have already been shown to be heritabl [21], they are prime candidates to act as endophenotypes for establishing the connection between genotype and brain function.
